# Alzheimer’s Amyloid-β Accelerates Cell Senescence and Suppresses SIRT1 in Human Neural Stem Cells

**DOI:** 10.3390/biom14020189

**Published:** 2024-02-04

**Authors:** Rongyao Li, Yi Li, Haowei Zuo, Gang Pei, Shichao Huang, Yujun Hou

**Affiliations:** 1 Institute for Regenerative Medicine, Shanghai East Hospital, Shanghai Key Laboratory of Signaling and Disease Research, Frontier Science Center for Stem Cell Research, School of Life Sciences and Technology, Tongji University, Shanghai 200092, China; lirongyao2022@sibcb.ac.cn (R.L.); yi.li@sibcb.ac.cn (Y.L.); 2031528@tongji.edu.cn (H.Z.); 2 State Key Laboratory of Cell Biology, CAS Center for Excellence in Molecular Cell Science, Shanghai Institute of Biochemistry and Cell Biology, Chinese Academy of Sciences, University of Chinese Academy of Sciences, Shanghai 200031, China; 3 The First Affiliated Hospital, Zhejiang University School of Medicine, and Liangzhu Laboratory of Zhejiang University, Hangzhou 310000, China; 4 Shanghai Key Laboratory of Signaling and Disease Research, Collaborative Innovation Center for Brain Science, School of Life Sciences and Technology, Tongji University, Shanghai 200092, China; 5 Institute for Stem Cell and Regeneration, Chinese Academy of Sciences, Beijing 100100, China

**Keywords:** Aβ, cell senescence, neural stem cells, SIRT1, ROS

## Abstract

As a lifelong source of neurons, neural stem cells (NSCs) serve multiple crucial functions in the brain. The senescence of NSCs may be associated with the onset and progression of Alzheimer’s disease (AD). Our study reveals a noteworthy finding, indicating that the AD-associated pathogenic protein amyloid-β (Aβ) substantially enhances senescence-related characteristics of human NSCs. These characteristics encompass the enhanced expression of p16 and p21, the upregulation of genes associated with the senescence-associated secretory phenotype (SASP), increased SA-β-gal activity, and the activation of the DNA damage response. Further studies revealed that Aβ treatment significantly downregulates the SIRT1 protein which plays a crucial role in regulating the aging process and decreases downstream PGC-1α and FOXO3. Subsequently, we found that SIRT1 overexpression significantly alleviates a range of Aβ-induced senescent markers in human NSCs. Taken together, our results uncover that Aβ accelerates cellular senescence in human NSCs, making SIRT1 a highly promising therapeutic target for senescent NSCs which may contribute to age-related neurodegenerative diseases, including AD.

## 1. Introduction

Neural stem cells (NSCs) are a type of multipotent stem cell that can differentiate into various types of neural cells, such as neurons, astrocytes, and oligodendrocytes [1]. NSCs are present in the developing and adult mammalian central nervous system (CNS) [2]. Neural stem cells play an essential role in the development and repair of the nervous system, as they can generate new neurons and glial cells to replace damaged or lost ones in the hippocampus [3]. However, with age, the self-renewal and differentiation abilities of neural stem cells gradually weaken [4,5]. This results in a decline in the quantity and quality of neural stem cells, affecting the regeneration and repair of the nervous system.

Aging has a significant and detrimental impact on the functioning of the brain [6]. Cellular senescence is one of the hallmarks of aging. Senescent cells usually show increased senescence-associated β-galactosidase (SA-β-gal) expression, cell cycle arrest, the secretion of senescence-associated secretory phenotype (SASP), increased reactive oxygen species (ROS) production, and an activated DNA damage response [7]. Alzheimer’s disease (AD) is an age-related neurodegenerative disorder. Senescent phenotypes have been found to be significantly upregulated in cell types associated with AD, including astrocytes, microglia, neuron, endothelial cells and oligodendrocyte progenitor cells [8,9,10]. These results indicated that cell senescence may be a potential target for therapy in AD.

Senescent NSCs accumulate in the aging brain and may impair neurogenesis and cognitive function. Studies report that the loss of Rack1 reduces neurogenesis and promotes cellular senescent phenotypes in NSCs [11]. Patients with AD often develop cognitive and memory impairments, which may be associated with the aging and loss of function of neural stem cells [12,13]. Using the senolytic compound ABT-263 to clear senescent neural stem cells in the hippocampus can induce stem cell proliferation and neurogenesis, supporting the significant role of stem cell senescence in promoting disease occurrence [14]. Therefore, it is highly promising to prevent neural stem cell senescence to ameliorate neural damage and treat neurodegenerative diseases associated with AD.

The sirtuin family consists of seven NAD^+^-dependent deacetylases (SIRT1-7) that regulate various cellular processes, such as metabolism, DNA repair, inflammation, and stress response [15]. Among them, SIRT1 is the most extensively studied for its role in resisting cellular senescence [16,17]. Abundant evidence suggests that downregulation of SIRT1 significantly accelerates cell senescence in various cell types, including endothelial cells, human mesenchymal stem cells (hMSCs), and primary human lung fibroblasts [18,19,20,21]. In contrast, restoring SIRT1 has been shown to alleviate cellular senescence phenotypes in multiple cellular models [21,22]. Additionally, the downstream target of SIRT1, PGC-1α, also contributes to the protection of mitochondrial function and anti-aging [23]. The role of SIRT1 in AD has also been discovered. There is growing evidence that SIRT1 plays a role in many processes in the onset and progression of AD, such as APP processing, neuroinflammation, and mitochondrial dysfunction [24,25]. And meanwhile, SIRT1 also inhibits the tau-related AD phenotype [26,27]. However, the role of SIRT1 in human neural stem cell senescence remains poorly studied in AD cell models. In our study, we found that Aβ, the major causative protein of AD, accelerates cellular senescence in human NSCs, accompanied by a significant downregulation of SIRT1 protein expression. Moreover, the overexpression of SIRT1 markedly alleviated the Aβ-induced cellular senescence phenotype, indicating that SIRT1 may be a promising target for anti-aging in neural stem cells.

## 2. Material and Methods

### 2.1. Cell Culture

Human induced pluripotent stem cell (iPSC)-derived NSCs (66-year-old healthy female (3L); 0-year-old healthy female (13A)) were provided by IxCell Biotechnology (Shanghai, China). [28]. Human NSCs were cultured in 50% DMEM/F12 (Gibco, New York, NY, USA) and 50% Neurobasal-A (Gibco) medium, containing 1 × MEM non-essential amino acids solution, 1 × N2 supplement (Gibco), 1 × GlutaMAX (Gibco), 1 × B27 supplement (Gibco), 10 ng/mL bFGF, 10 ng/mL hlif, 3 μM CHIR99021 (Selleckchem, Shanghai, China), 5 μM SB431542 (Selleckchem), and 200 μM ascorbic acid (Sigma-Aldrich, St. Louis, MO, USA). Cells were incubated at 37 °C in a humidified atmosphere of 5% CO_2_/95% air (*v*/*v*).

### 2.2. Lentivirus Preparation and Infection

Lentivirus was prepared as previously reported [29]. In brief, we transfected HEK293T cells with psPAX2 (15 µg), pMD2.G (10 µg), and FUGW-SIRT1 (20 µg) plasmids using polyethyleneimine (PEI). After transfection, the culture medium was replaced six hours later, and the lentiviral supernatant was collected 72 h later. We filtered the supernatant through 0.45 µm filters to remove cell debris and concentrated the virus by ultracentrifugation at 27,000× *g* for 2 h. We then resuspended the virus pellets in 1× PBS for infection. Then, we infected human neural stem cells in suspension by adding concentrated lentiviruses to the medium containing 8 µg/mL polybrene (Sigma-Aldrich) and incubating for 1 h.

### 2.3. Aβ_42_ Oligomers Preparation

We prepared Aβ_42_ oligomers (Aβ) according to previous publications [30]. In brief, 2 mg of Aβ_1–42_ peptide (AMYD-003, supplied by CHINESE PEPTIDE, Hangzhou, China) was dissolved in 2 mL of chilled hexafluoroisopropanol (HFIP from Sigma-Aldrich). This solution was then transferred to Protein LoBind tubes (030108094, Eppendorf, Hamburg, Germany) and left to air-dry overnight at room temperature. After the HFIP treatment, the Aβ_1–42_ peptides were resuspended in dimethyl sulfoxide (DMSO) and diluted in phenol red-free DMEM/F12 medium, resulting in stock solutions with a concentration of 100 μM. Aβ_42–1_ (P9005, Beyotime, Shanghai, China) was used as a negative control. In this paper, Aβ presented Aβ_1–42_.

### 2.4. siRNA Transfection

The knockdown of SIRT1 was conducted via the transfection of specific siRNA using lipofectamine 3000 (Invitrogen, Carlsbad, CA, USA) according to the manufacturer’s instructions, as we previous reported [31].

The siRNA primers were as follows:

siSIRT1: Forward 5′-CACCUGAGUUGGAUGAUAUTT-3′

Reverse 5′-AUAUCAUCCAACUCAGGUGTT-3′.

### 2.5. Senescence-Associated β-Galactosidase (SPiDER-βGal) Staining Assay

SA-β-gal staining was conducted by using SPiDER-βGal (Dojindo, Kumamoto, Japan) according to the manufacturer’s instructions. First, cells were incubated with Bafilomycin A1 working solution for 1 h. Then, cells were incubated with SPiDER-βGal working solution for 30 min at 37 °C. After washing twice with PBS, the samples were fixed for 15 min at room temperature with 4% PFA. Fluorescent images were captured using the Olympus FV3000 Laser scanning confocal microscope.

### 2.6. Western Blot

After the aforementioned treatments, we separated the total cell lysates using either 12.5% or 10% sodium dodecyl sulfate–polyacrylamide gel electrophoresis (SDS-PAGE). These were then transferred to nitrocellulose membranes (at a constant current of 400 mA for 2 h at 4 °C). We then blocked the membranes with 5% nonfat milk in TBS that contained 0.1% Tween-20. In the next step, the membranes were incubated overnight at 4 °C with the designated primary antibodies and followed by incubation with the corresponding horseradish peroxidase (HRP)-conjugated secondary antibody. The membranes were visualized using an enhanced chemiluminescence (ECL) substrate kit (Bio-Rad, Hercules, CA, USA) with the ECL detection system (Sage Creation Science, Beijing, China). Quantification of the Western blot bands was carried out using ImageJ software (version 1.52). The antibodies used were as follows: anti-p16 (1:1000, 18769S, Cell Signaling Technology, Danvers, MA, USA), anti-p21 (1:1000, 2947S, Cell Signaling Technology), anti-γH2AX (1:1000, 9718, Cell Signaling Technology), anti-SIRT1 (1:1000, 8469, Cell Signaling Technology), PGC-1α (1:1000, 66369-1-Ig, Proteintech, Wuhan, China) and actin (1:1000, A2066, Sigma-Aldrich).

### 2.7. Immunofluorescence Staining

In brief, after the indicated treatments, the cells were subjected to a 15 min treatment with 4% paraformaldehyde at room temperature. Subsequently, the cells were exposed to primary antibodies that were appropriately diluted in a permeabilization and blocking buffer consisting of 3% donkey serum and 0.3% Triton-X 100 in phosphate-buffered saline (PBS) overnight at 4 °C. Following a minimum of three washes with PBS, the cells were incubated with secondary antibodies conjugated with fluorescent markers, which were suitably diluted in the aforementioned permeabilization and blocking buffer, for a duration of 2 h at room temperature. This was followed by a 15 min staining process with DAPI (1:3000, Beyotime). The slides were then mounted, and images were captured using the Olympus FV3000 microscope. The antibodies used were anti-γH2AX (1:800, 9718, Cell Signaling Technology) and anti-8-OHdG (1:1000, 200-301-A99, Rockland, Limerick, PA, USA).

### 2.8. Reverse Transcription and Quantitative Real-Time PCR

After treatment, the total RNA was isolated using TRIzol Reagent (TaKaRa, Japan) following the manufacturer’s instructions. RNA was then reverse-transcribed into cDNA using the Evo M-MLV RT Premix kit (Accurate Biology, Changsha, China). Quantitative real-time PCR was performed on the LightCycler 96 qPCR system (Roche, Basel, Switzerland) using the Taq Pro Universal SYBR qPCR Master Mix (Vazyme Biotech, Nanjing, China).

The primers used were as follows:

*p21*, Forward: 5′-CGATGGAACTTCGACTTTGTCA-3′, Reverse: 5′- GCACAAGGGTACAAGACAGTG-3′

*p16*, Forward: 5′-GGGTTTTCGTGGTTCACATCC-3′, Reverse: 5′-CTAGACGCTGGCTCCTCAGTA-3′

*MMP3*, Forward: 5′-CTGCTGTTGAGAAAGCTCTG-3′, Reverse:5′- AATTGGTCCCTGTTGTATCCT-3′

*PAI-1*, Forward: 5′-ACCGCAACGTGGTTTTCTCA-3′, Reverse: 5′- TTGAATCCCATAGCTGCTTGAAT-3′

*p53*, Forward: 5′-CCCCTCCTGGCCCCTGTCATCTTC-3′, Reverse: 5′- GCAGCGCCTCACAACCTCCGTCAT-3′

*Δ133p53*, Forward: 5′-TGACTTTCAACTCTGTCTCCTTCCT-3′, Reverse: 5′- GGCCAGACCATCGCTATCTG-3′

*LaminB1*, Forward: 5′-GTATGAAGAGGAGATTAACGAGAC-3′, Reverse: 5′- TACTCAATTTGACGCCCAG-3′

*HPRT*, Forward: 5′-CCTGGCGTCGTGATTAGTGAT-3′, Reverse: 5′-AGAC GTTCAGTCCTGTCCATAA-3′.

### 2.9. Measurement of Intracellular ROS Generation

Intracellular ROS production was detected using the ROS Assay Kit (Beyotime, S0033). Briefly, cells were initially seeded in a 96-well plate at a density of 1 × 104 cells/well and subsequently treated with or without 10 μM Aβ. After completion of the treatment, the cells were co-stained with 10 μM DCFH-DA and 3 μg/mL Hoechst (Beyotime, C1022) at 37 °C for 20 min. Then, cells were washed twice with PBS, and the ROS levels were determined using BioTek SynergyNEO (BioTek, Winusky, VT, USA) at excitation/emission wavelengths of 488/525 nm for DCFH-DA and 350/461 nm for Hoechst. Alternatively, the cells in the 96-well black plate were observed using a laser-scanning confocal microscope (Operetta, Perkin Eimer, Waltham, MA, USA).

### 2.10. Mitochondrial ROS Detection

In this study, cells were seeded in a 96-well plate at 1 × 10^4^ cells per well and treated with or without 10 μM Aβ for 48 h. After treatment, cells were co-stained with 2.5 μM MitoSOX Red mitochondrial superoxide indicator (M36008, Invitrogen) and 3 μg/mL Hoechst for 20 min at 37 °C. The fluorescent signals were recorded using BioTek SynergyNEO at excitation/emission wavelengths of 510/580 nm for MitoSOX and 350/461 nm for Hoechst. The MitoSOX fluorescence intensity was normalized to the Hoechst.

### 2.11. Statistical Analysis

The data analysis was conducted using Prism 8.0. Quantitative data were reported as mean ± SEM. The unpaired Student’s *t*-test (two-tailed) was employed to compare two datasets, while the one-way analysis of variance (ANOVA) was utilized for comparisons involving more than two datasets or groups. Statistical significance was considered at *p* < 0.05.

## 3. Results

### 3.1. Amyloid-β Upregulated Senescent Markers in Human NSCs

**Figure 1:**
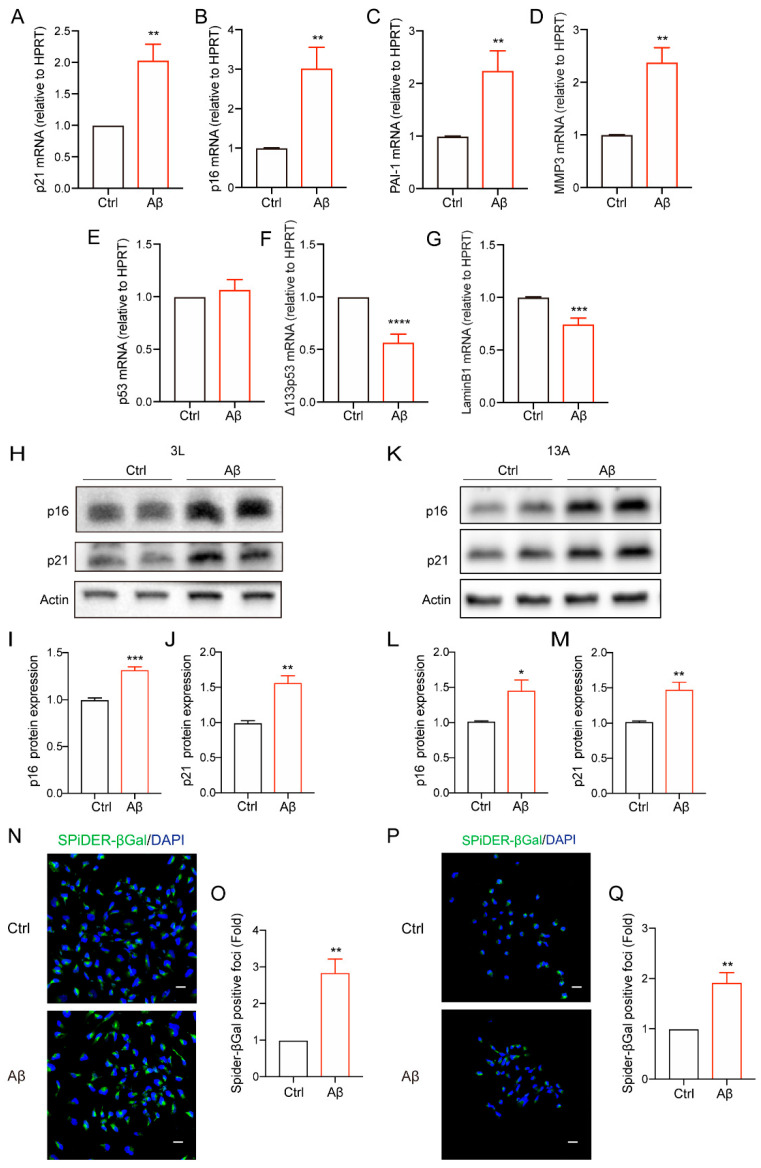
Amyloid-β upregulated senescent markers in human NSCs. (**A**–**G**) *p21* (**A**), *p16* (**B**), *PAI-1* (**C**), *MMP3* (**D**), *p53* (**E**), *Δ133p53* (**F**) and *LaminB1* (**G**) mRNA levels were analyzed at 72 h after Aβ (5 µM) treatment. (**H**) Cells were treated with Aβ (5 µM) for 72 h in 3L NSC, and the levels of p16 and p21 were analyzed using Western blotting. (**I**,**J**) Quantification of relative p16 (**I**), p21 (**J**) protein levels in (**H**). (**K**) Cells were incubated with Aβ (5 µM) for 72 h in 13A NSC, and the levels of p16 and p21 were analyzed using Western blotting. (**L**,**M**) Quantification of relative p16 (**L**), p21 (**M**) protein levels in (**K**). (**N**) The representative images of SA-β-gal staining in 3L NSC treated with or without Aβ (5 μM) for 72 h. n = 3, scale bar, 20 μm. (**O**) Quantitation of (**N**). (**P**) The representative images of SA-β-gal staining in 13A NSC treated with or without Aβ (5 μM) for 72 h. n = 3, scale bar, 20 μm. (**Q**) Quantitation of (**P**). The data were presented as mean ± SEM, n ≥ 3 independent experiments, * *p* < 0.05, ** *p* < 0.01, *** *p* < 0.001, and **** *p* < 0.0001, analyzed using unpaired Student’s *t*-test (two-tailed). (Original western blot images can be found in Supplementary Materials Excel File S1).

According to current research, cellular senescence is characterized by cell cycle arrest, increased senescence-associated secretory phenotypes (SASP), the age-related upregulation of β-galactosidase activity, and the activation of the DNA damage response [32,33]. First, by examining the expression of a series of age-related gene expressions, we found that Aβ significantly upregulated the cell cycle-related genes, including *p21* and *p16* (Figure 1A,B), as well as the age-related secreted proteins *PAI-1* and *MMP3* in human NSCs (Figure 1C,D), which were characterized via staining with the stem cell marker-expressed Nestin and Sox2 (Figure S1). At the same time, it was also observed that Aβ had no obvious effect on *p53* (Figure 1E), but the expression of its subunit *Δ133p53*, an inhibitory isoform of p53, which has been reported to delay cellular aging [34,35], was significantly reduced (Figure 1F). Additionally, a reduction in *laminB1* (Figure 1G), an important component of the nuclear scaffold [36] that has been studied as a senescence-associated biomarker in many studies [36,37,38,39], was observed due to Aβ stimulation. To further validate, Aβ significantly increased the protein expression of p16 and p21 in both types of human neural stem cells (3L and 13A, Figure 1H–M). The Aβ_42–1_, as a negative control, had no obvious effect (Figure S2). Furthermore, by using SPiDER-βGal fluorescent staining, it was found that Aβ also significantly increased the activity of SA-β-gal (Figure 1N–Q). Taken together, these results indicate that Aβ can accelerate the aging process of neural stem cells.

### 3.2. Amyloid-β Promoted ROS Production and Senescence-Associated DNA Damage

**Figure 2:**
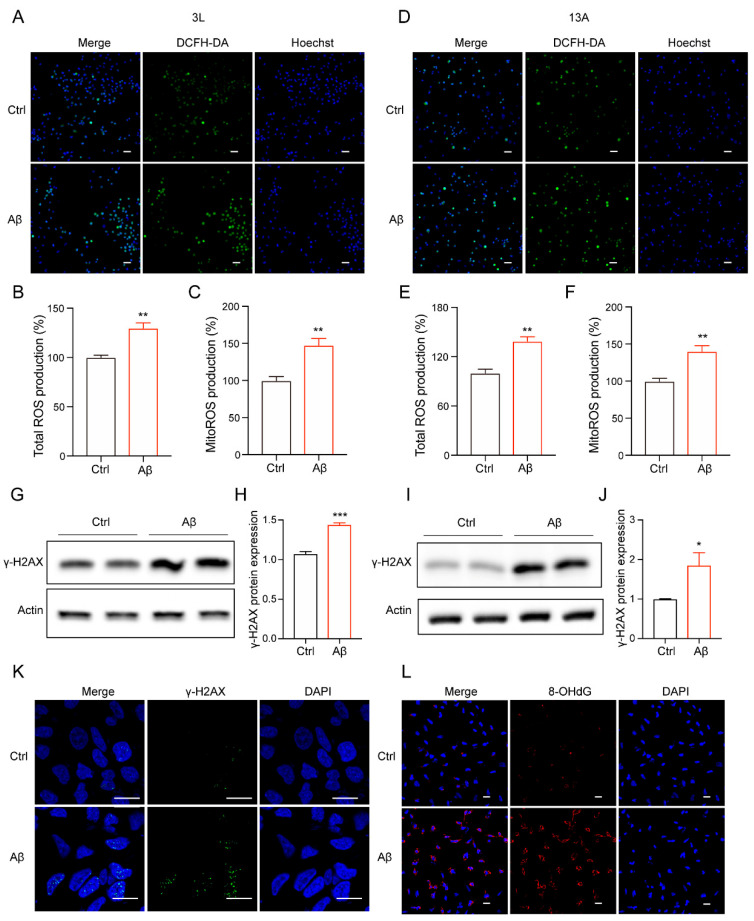
Amyloid-β promoted ROS production and senescence-associated DNA damage. (**A**) The representative images of 3L NSC treated with Aβ (5 μM) for 48 h, then co-stained with DCFH-DA and Hoechst. The pictures were obtained via Operetta. Scale bars, 50 μm. (**B**) The quantification of (**A**), showing relative ROS generation of cells treated with Aβ (5 μM) for 48 h. (**C**) Mitochondrial ROS production in the cells treated with Aβ (5 μM) for 48 h. The signal of MitoSOX was normalized to Hoechst. (**D**) The representative images of 13A NSC treated with Aβ (5 μM) for 48 h, then co-stained with DCFH-DA and Hoechst. The pictures were obtained via Operetta. Scale bars, 50 μm. (**E**) The quantification of (**A**), showing relative ROS generation of cells treated with Aβ (5 μM) for 48 h. (**F**) Mitochondrial ROS production in the cells treated with Aβ (5 μM) for 48 h. The signal of MitoSOX was normalized to Hoechst. (**G**) Western blot analysis was performed to detect γ-H2AX. 3L NSC were treated with Aβ (5 μM) for 72 h. (**H**) The quantification of (**G**). (**I**) Western blot analysis was performed to detect γ-H2AX. 13A NSC were treated with Aβ (5 μM) for 72 h. (**J**) The quantification of (**I**). (**K**) Representative images of γ-H2AX staining in 3L NSC treated by Aβ (5 µM) at 72 h. The pictures were obtained via Olympus FV3000. Scale bar, 20 µm. (**L**) Representative images of 8-OHDG staining in 3L NSC treated with Aβ (5 µM) at 72 h. The pictures were obtained via Olympus FV3000. Scale bar, 20 µm. The data were presented as mean ± SEM, n ≥ 3 independent experiments, * *p* < 0.05, ** *p* < 0.01, *** *p* < 0.001, analyzed using unpaired Student’s *t*-test (two-tailed). (Original western blot images can be found in Supplementary Materials Excel File S1).

Oxidative stress occurs due to an increase in ROS production or a decrease in antioxidant capacity in AD. Oxidative damage is also a significant contributor to cellular senescence [40,41]. Aβ can accumulate in mitochondria and impair their respiratory chain complexes, resulting in electron leakage and subsequent ROS generation [42]. And, Aβ can also impair antioxidant defense mechanisms [43]. In this paper, we found that Aβ significantly increased the levels of reactive oxygen species (ROS) in both types of neural stem cells using DCFH-DA staining, indicating the accumulation of oxidative damage (Figure 2A,B,D,E). Mitochondria are known to generate 90% of cellular reactive oxygen species [44,45]. Therefore, we assessed ROS levels in mitochondria and observed a similar increase in ROS production upon exposure to Aβ (Figure 2C,F). Due to the high-glucose medium, NSCs may rely mainly upon glycolysis in our condition. Therefore, we hypothesized that Aβ may directly interfere with the mitochondria antioxidant defense systems or indirectly influence mitochondrial proteins, reducing the ability to clear ROS effectively. The accumulation of ROS can affect the integrity of genomic DNA. Via immunoblotting experiments, we observed that Aβ upregulated the phosphorylation of histone H2AX, also known as γ-H2AX, in both neural stem cells (Figure 2G–J). Then, we detected, via immunofluorescence staining, that Aβ induced more γ-H2AX foci compared with the untreated control (Figure 2K). Furthermore, we found that Aβ significantly elevated the levels of 8-hydroxy-2′-deoxyguanosine (8-OHdG), a marker of oxidative DNA damage (Figure 2L). Collectively, these results provide evidence that Aβ induces the accumulation of oxidative damage in neural stem cells and senescence-associated DNA damage and accelerated cellular senescence.

### 3.3. SIRT1 Was Decreased in Amyloid-β-Induced Cell Senescence in Human NSCs

**Figure 3:**
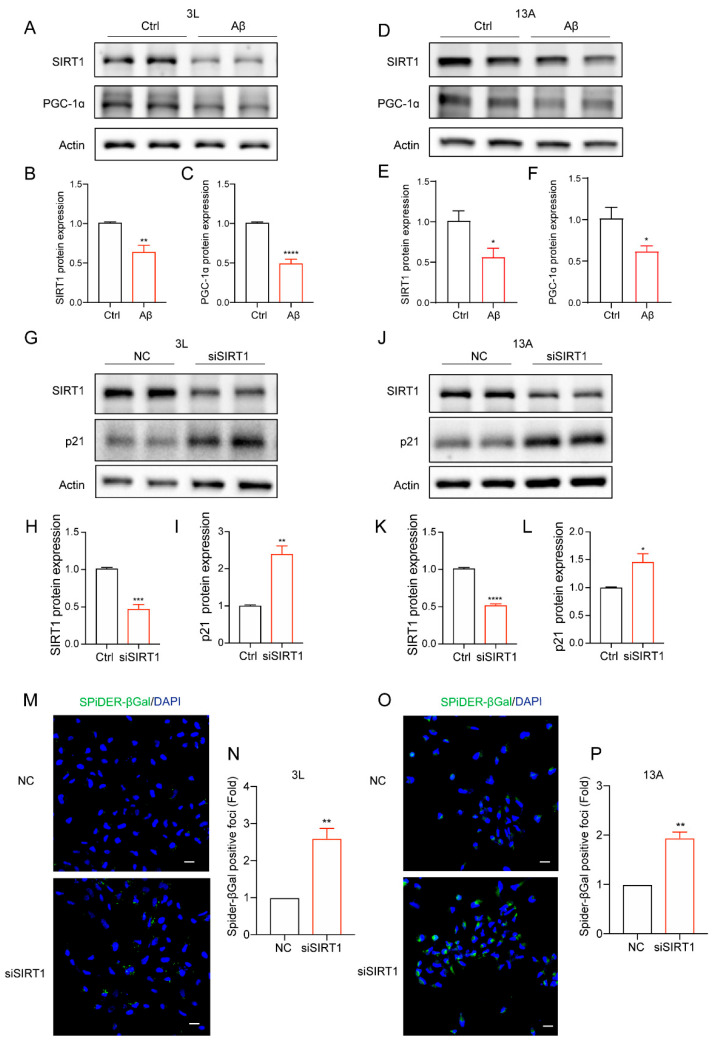
SIRT1 was decreased in amyloid-β-induced cell senescence in human NSCs. (**A**) Western blot analysis of SIRT1 and PCG-1α protein expression after treatment with Aβ (5 µM) in 3L NSC. (**B**,**C**) Quantification of SIRT1 (**B**) and PCG-1α (**C**) protein levels in (**A**). (**D**) Western blot analysis of SIRT1 and PCG-1α protein expression after treatment with Aβ (5 µM) in 13A NSC. (**E**,**F**) Quantification of SIRT1 (**E**) and PCG-1α (**F**) protein levels in (**D**). (**G**) The cells were subjected to transfection with a negative control (NC) and siSIRT1 in 3L NSC. After 72 h, these cells were then harvested. SIRT1 and p21 levels were analyzed with Western blotting. (**H**,**I**) Quantification of SIRT1 (**H**) and p21 (**I**) protein levels in 3L NSC. (**J**) The cells were subjected to transfection with a negative control (NC) and siSIRT1 in 13A NSC. After 72 h, these cells were then harvested. SIRT1 and p21 levels were analyzed with Western blotting. (**K**,**L**) Quantification of SIRT1 (**K**) and p21 (**L**) protein levels in 13A NSC. (**M**) The representative images of SA-β-gal staining in 3L NSC following NC or siSIRT1 treatment after 72 h. (**N**) The quantification analysis of relative Spider-βGal-positive foci in (**M**). (**O**) The representative images of SA-β-gal staining in 13A NSC following NC or SiSIRT1 treatment after 72 h. (**P**) Quantification of relative Spider-βGal-positive foci in (**O**). The data were presented as mean ± SEM, n ≥ 3 independent experiments, * *p* < 0.05, ** *p* < 0.01, *** *p* < 0.001, and **** *p* < 0.0001, analyzed using unpaired Student’s *t*-test (two-tailed). (Original western blot images can be found in Supplementary Materials Excel File S1).

Members of the sirtuin family play a crucial role in combating cellular senescence [46]. According to our previous reports, SIRT1 is involved in regulating the process of neuronal senescence [31]. As a downstream regulator of SIRT1, PGC-1α plays an important role in mitochondrial biogenesis, glucose metabolism regulation, antioxidant response and lipid metabolism regulation [47,48,49]. Another downstream regulator FOXO3 also plays an important role in cell senescence [50,51]. The SIRT1/PGC-1α pathways have been proved to participate in cellular senescent processes [52]. To further explore the role of SIRT1 in neural stem cell senescence, we found that Aβ significantly downregulated the protein expression levels of SIRT1 and its downstream protein PGC-1α (Figure 3A–F) and FOXO3 (Figure S4) in human NSCs via immunoblotting. The results suggest that SIRT1 may be involved in regulating the senescence of neural stem cells.

In addition, we decreased SIRT1 expression by targeting the transcriptional level of SIRT1 and observed a significant decrease in the protein level of SIRT1 via immunoblotting experiments (Figure 3G,H,J,K). Moreover, we found that the downregulation of SIRT1 by transfecting siSIRT1 significantly increased the protein level of p21 (Figure 3G,I,J,L). Meanwhile, compared to the negative control, siSIRT1 treatment obviously increased SPiDER-βGal positive foci (Figure 3M–P), suggesting the upregulation of SA-β-gal activity. Furthermore, Aβ upregulated the p21 protein level in which SIRT1 had been knocked down (Figure S3). These results imply that SIRT1 may be associated with the senescence of human neural stem cells, and Aβ may accelerate cell senescence in human NSCs by modulating SIRT1.

### 3.4. The Recovery of SIRT1 Rescued Cellular Senescence in Human NSCs

**Figure 4:**
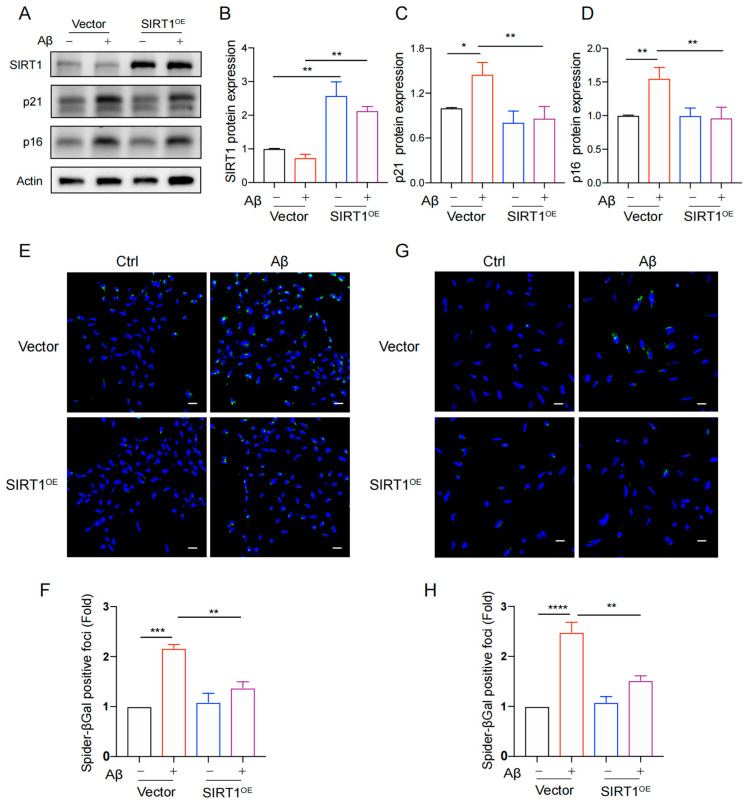
Overexpression of SIRT1 rescued Aβ-induced cell senescence. (**A**) The 3L NSC cells were subjected to infection with either SIRT1 lentivirus or vector, followed by incubation with or without Aβ (5 µM) for a duration of 72 h. Subsequently, the protein levels of SIRT1, p21, and p16 were analyzed using Western blot analysis. (**B**–**D**) Quantification of SIRT1 (**B**), p21 (**C**), and p16 (**D**) protein levels in (**A**). (**E**) The representative images show SA-β-gal staining in 3L NSC treated with Aβ or without for 72 h, following infection with lentivirus of SIRT1 or vector. (**F**) Quantification of relative Spider-βGal-positive foci in (**E**). (**G**) The representative images show SA-β-gal staining in 13A NSC treated with Aβ or without for 72 h, following infection with lentivirus of SIRT1 or vector. (**H**) Quantification of relative Spider-βGal-positive foci in (**G**). The pictures were obtained by Olympus FV3000. Scale bar, 20 µm. The data were presented as mean ± SEM, n ≥ 3 independent experiments, * *p* < 0.05, ** *p* < 0.01, *** *p* < 0.001, and **** *p* < 0.0001, analyzed by two-way ANOVA. (Original western blot images can be found in Supplementary Materials Excel File S1).

Based on the above results, we used lentivirus to overexpress SIRT1 in human NSCs. The successful overexpression of SIRT1 was verified by Western blot analysis (Figure 4A,B). Subsequently, it was observed that SIRT1 overexpression significantly downregulated the protein expression of p21 and p16 compared with the control group (Figure 4A,C,D). Furthermore, using SPiDER-βGal staining, we observed a significant decrease in SA-β-galactosidase activity by the overexpression of SIRT1 compared with the control group (Figure 4E–H). We validated these results in another NSC cell type (Figure S5A–C). SIRT1 overexpression rescued the high expression of γ-H2AX protein induced by Aβ in 3L NSC and 13A NSC (Figure S5C,D). These results revealed that the overexpression of SIRT1 can significantly alleviate Aβ-induced neural stem cell senescence and senescence-associated DNA damage, and targeting the upregulation of SIRT1 may potentially serve as a feasible strategy to mitigate neural stem cell senescence.

## 4. Discussion

Neural stem cells (NSCs) are found in the subgranular zone (SGZ) of the hippocampus and the subventricular zone (SVZ) of the lateral ventricles and maintain the ability to self-renew and differentiate into new neurons and glial cells [53]. The cumulative burden of age-related neurodegenerative diseases, such as AD, leads to brain degeneration, significantly affecting the regenerative potential of the neural stem cell niche [6,54]. The accumulation of senescent neural precursor cells decreased adult neurogenesis in the hippocampus with age in mice [14]. Alzheimer’s disease is an age-related neurodegenerative disease, including defective neurogenesis [55,56,57,58]. Defective neurogenesis causes a reduction in new-born neurons, potentially leading to the impairment of cognitive function [59].

In AD patients, the deposition of Aβ is usually found and recognized as one of the hallmarks of AD [60,61]. The role of Aβ in the pathogenesis of AD has been extensively studied. However, whether it affects human NSC senescence is rarely studied. In this study, we evaluated the impact of AD-associated Aβ on human iPSC-derived NSCs and found that Aβ significantly upregulated the senescence phenotypes in human NSCs, including upregulating the expression of p16 and p21, increasing SA-β-gal activity, and activating the DNA damage response. Our results indicated that Aβ, as the core pathology protein, accelerates NSC senescence more than the normal aging speed, thus impairing neurogenesis and aggravating disease pathogenesis. Certainly, other factors associated with AD, such as tau pathology, inflammation, and APOE4 mutations, may also contribute to neural stem cell senescence.

Sirtuins are a family of proteins involved in multiple cellular processes associated with aging, including DNA damage, oxidative stress, and mitochondrial dysfunction [62,63,64]. The sirtuin family has beneficial effects on AD [65,66,67]. Among them, SIRT1 is involved in regulating neural stem cell function, affecting self-renewal, differentiation, epigenetic regulation, and the aging processes [68,69]. However, further studies are needed to determine the involvement of SIRT1 in the senescent process of human NSCs in AD. Here, our results demonstrate that Aβ treatment reduced the expression level of SIRT1 in human NSCs, while the increase in SIRT1 expression efficiently rescued the Aβ-induced senescent phenotype of human NSCs. These findings suggest that SIRT1 may be involved in regulating Aβ-accelerated NSC senescence. Some clinically and preclinically used drugs, such as metformin and resveratrol, have anti-aging effects by modulating SIRT1 [70,71]. Anti-aging interventions by modulating SIRT1 may also be used to slow NSC aging in AD. Only two iPSC-derived NSCs were used in our study, and more samples should be conducted for verification. The relationship between senescent NSCs and NSC neuro-genic differentiation need more research in the future. Furthermore, our work was conducted in vitro, and more in vivo models are needed for further validation.

## 5. Conclusions

In summary, our study revealed that the AD-associated pathogenic protein Aβ accelerated cellular senescence in human NSCs via SIRT1 inhibition, providing a hopeful target for anti-aging in neural stem cells and the treatment of age-related neurodegenerative diseases.

## Data Availability

All data used to support the findings of this study are included within the article.

## Supplementary Materials

The following supporting information can be downloaded at: https://www.mdpi.com/article/10.3390/biom14020189/s1, Figure S1: Representative images of immunofluorescent staining in 3L NSC using Sox2, Nestin, and DAPI. Figure S2: The Aβ_42-1_ did not affect p21 protein expression in human neural stem cells. Figure S3: Aβ accelerates the effects of SIRT1 knockdown to upregulated p21 protein level. Figure S4: Aβ downregulated the protein expression levels of SIRT1 and its downstream protein FOXO3 in 3L NSC. Figure S5: Overexpression of SIRT1 rescued Aβ-induced cell senescent markers. Excel File S1: Raw data of Western blot images used in the manuscript. Excel File S2: Raw data of Western blot images used for the quantifications.
