# Supplementary material for: Alzheimer’s Amyloid-β Accelerates Cell Senescence and Suppresses SIRT1 in Human Neural Stem Cells
Source: Biomolecules. 2024 Feb 4;14(2):189. doi: 10.3390/biom14020189 (PMC10886734; doi:10.3390/biom14020189)
Supplement: Supplementary file 1 [file biomolecules-14-00189-s001.zip › Supplementary Figures.pdf]

Article

Alzheimer's Amyloid-β Accelerates Cell Senescence and Suppresses SIRT1 in Human Neural Stem Cells

Rongyao Li [1,2,†], Yi Li [1,2,3,†], Haowei Zuo [1], Gang Pei [2,4,5], Shichao Huang [2,*] and Yujun Hou [1,*]

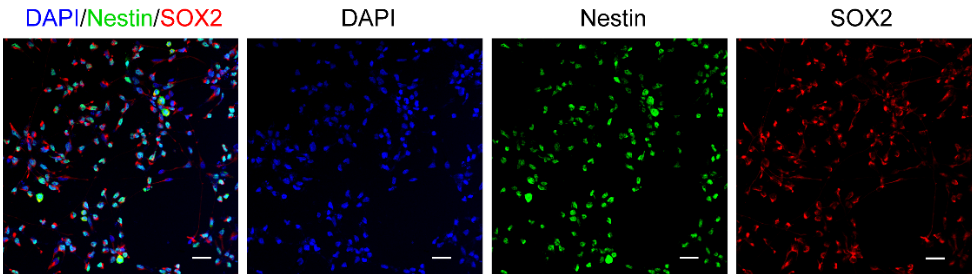

Figure S1. Representative images of immunofluorescent staining in 3L NSC using Sox2, Nestin and DAPI. Scale bar: 100 μm.

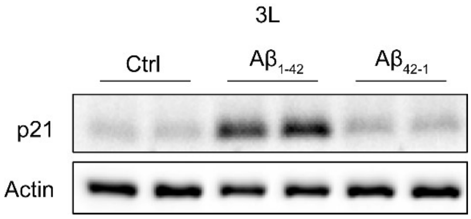

Figure S2. The Aβ$_{42\text{-}1}$ had no effect on p21 protein expression in human neural stem cells. Western blot analysis of p21 after treatment with Aβ$_{1\text{-}42}$ (5 μM) or Aβ$_{42\text{-}1}$ (5 μM) for 72 h in 3L NSC.

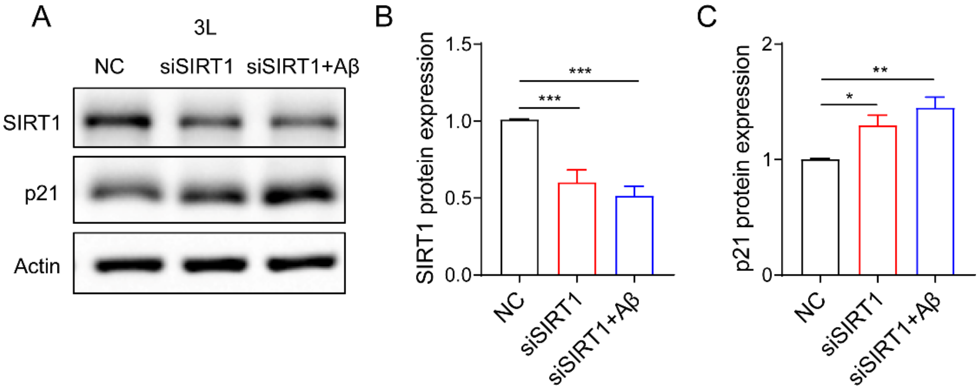

Figure S3. Aβ accelerates the effects of SIRT1 knockdown to upregulated p21 protein level. (**A**) 3L NSC were transfected with negative control (NC), siSIRT1 or siSIRT1 + Aβ (5 μM), and harvested after 72 h. Western blot analysis of SIRT1 and p21 protein levels. (**B, C**) Quantification of SIRT1 (B) and p21 (C) protein levels in (A). The data were presented as mean ± SEM, n ≥ 3 independent experiments, $*p < 0.05$, $**p < 0.01$, $***p < 0.001$, analyzed by one-way ANOVA.

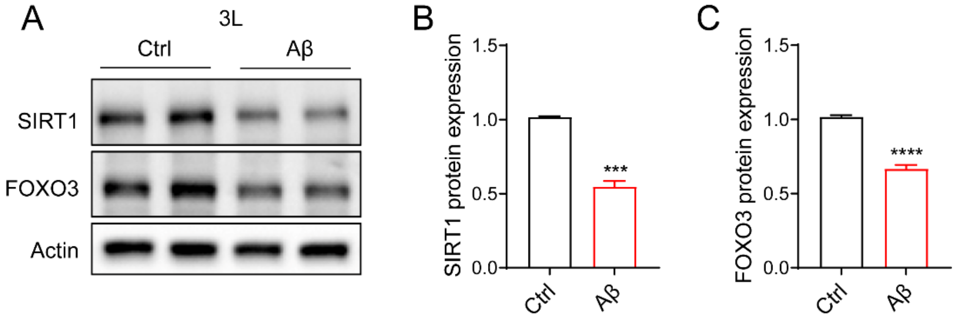

Figure S4. Aβ downregulated the protein expression levels of SIRT1 and its downstream protein FOXO3 in 3L NSC. (**A**) Western blot analysis of SIRT1 and FOXO3 protein expression after treatment with $A\beta_{1\text{-}42}$ (5 μM) in 3L NSC. (**B-C**) Quantification of SIRT1 (B) and FOXO3 (C) protein levels in (A). The data were presented as mean ± SEM, n ≥ 3 independent experiments, ***$p < 0.001$, and ****$p < 0.0001$, analyzed by unpaired Student's t-test (two-tailed).

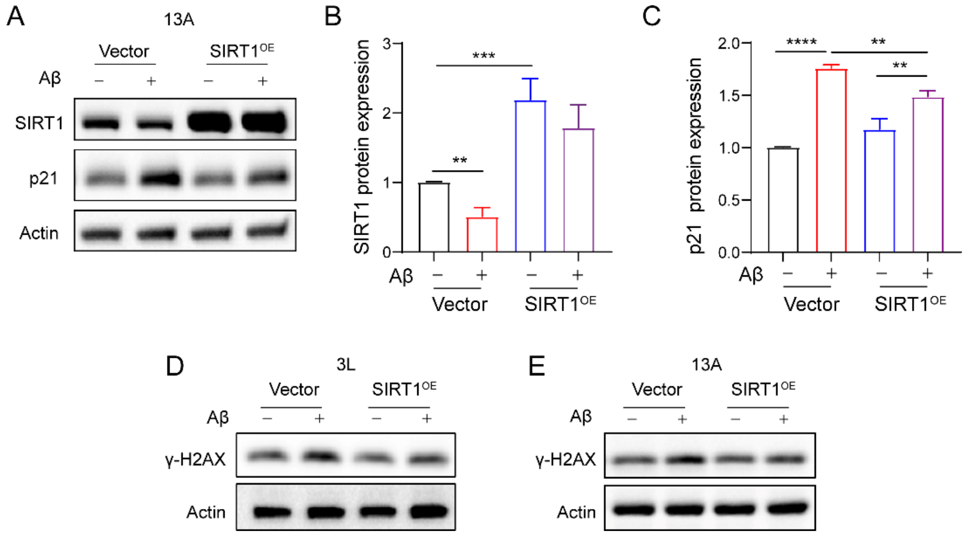

Figure S5. Overexpression of SIRT1 rescued Aβ-induced cell senescent markers. (**A**) Cells were infected with lentivirus of SIRT1 or vector in 13A NSC, and incubated with or without Aβ (5 μM) for 72 h. Western blot analysis of SIRT1 and p21 protein levels. (**B, C**) Quantification of SIRT1 (B) and p21 (C) protein levels in (A). (**D, E**) Cells were infected with lentivirus of SIRT1 or vector in 3L NSC (D) and 13A NSC (E), and incubated with or without Aβ (5 μM) for 72 h. Western blot analysis of γ-H2AX protein levels. The data were presented as mean ± SEM, n ≥ 3 independent experiments, **$p < 0.01$, ***$p < 0.001$, and ****$p < 0.0001$, analyzed by two-way ANOVA.
